# A review of the morphology of ohatsuki (stamen- or ovule-bearing) leaves, with relevance to physiological analyses of *Ginkgo biloba*

**DOI:** 10.3389/fpls.2026.1983013

**Published:** 2026-10-29

**Authors:** Yoshie Uchida, Hongqiao Lu, Sayumi Hotta, Hirofumi Yamashita, Masami Kobayashi, Hakuto Kageyama, Kazuhito Inoue, Hidenobu Uchida

**Affiliations:** 1 Department of Food Business, Nagoya Bunri University, Inazawa, Japan; 2 Graduate School of Environmental and Human Sciences, Meijo University, Nagoya, Japan; 3 Sobue Ginkgo biloba Research Laboratory, Inazawa, Japan; 4 Department of Information and Environmental Science, Kyoto Prefectural University, Kyoto, Japan; 5 Department of Material Science, University of Tsukuba, Tsukuba, Japan; 6 Research Institute for Integrated Science, Kanagawa University, Yokohama, Japan; 7 Department of Biochemistry and Biotechnology, Kanagawa University, Yokohama, Japan

**Keywords:** fossil, *Ginkgo biloba*, ohatsuki icho, stamen- or ovule-bearing leaves, stoma

## Abstract

A *Ginkgo biloba* variant in which either stamens or ovules are generated on the edge of the leaf is known as “ohatsuki icho”. In this review, we highlight the morphological similarities among ohatsuki leaves, reproductive organs, and the leaves and seeds of fossil plants. Although ohatsuki phenotypes appear to be controlled by sex-related bud sports, their expression seems to be only possible in the river system, in the presence of seasonal winds, suggesting water-related control. We discuss a previous report suggesting that water conductance may contribute to the occurrence of ohatsuki malformations in ohatsuki shoots and leaves, although a physiological evaluation of water transpiration in ohatsuki leaves has yet to be conducted. The information presented in this review may provide insights into the evaluation of the above-mentioned hypothesis and may identify key factors related to sexual evolution in land plants.

## 1 Introduction

*Ginkgo biloba* trees provide edible seeds and medicinal or insecticidal products (Hori et al., 1997; van Beek, 2000; Nagata, 2014; Yu et al., 2025). They are also used as street trees, as they are resistant to drought, exhaust gases, salt stress (Matsuura et al., 2026), and fire (Nagata, 2020). The long period of sexual maturation of female *G. biloba* trees (approximately 30 years) poses challenges for industrial use and genetic and horticultural studies. Because sexual reproduction involving sperm in *G. biloba* is unique (Hirase, 1896; Ikeno, 1906; Miyamura, 2020), morphological observations of the sexual organs have been conducted on this plant. A variety known as “ohatsuki icho”, which shows leaf malformation and produces stamens or ovules on the leaf edge (Shirai, 1891; Fujii 1896a, b, c; Sakisaka, 1926; Soma, 1997; Mochizuki, 2015), has been found in certain areas. Although Makino (1929) described the ohatsuki shoots as *Ginkgo biloba* L. var. *epiphylla* Makino, nov. var., Sakisaka (1926, 1958) regarded this as a forma whose morphology is dependent on the environment in its local habitat.

The responses of *G. biloba* trees to environmental stresses, such as salt or humidity (Matsuura et al., 2026), drought plus salt (Li et al., 2025), and salt plus melatonin (Yang et al., 2026), have been studied based on genome information (Liu et al., 2021). Stress responses have also been characterized at the stomatal level (Rudall et al., 2012), where water transpires (Carli et al, 2025; Matsuura et al., 2026).

Because of the morphological similarities between ovule-bearing ohatsuki leaves and the seeds of fossil ginkgo plants, ohatsuki malformations can be regarded as atavism. Zhou’s research group (Zhou and Zhang, 1989; Zheng and Zhou, 2004; Zhou et al., 2012) conducted a comprehensive taxonomic analysis of leaf- and seed-based species in fossil specimens collected from Yima (**Supplementary Material**), Henan Province, China (Crane, 2013; Crane and Shi, 2020). After ginkgo fossils collected in Japan were described by Yokoyama (1890), fossils of *Ginkgoidium* containing seeds and leaves were deposited in museums in Japan (Matsuoka, 2000; Yasuno, 2007).

In this review, we discuss the history of ohatsuki research, characterize the leaf and seed morphologies of ginkgo fossils, and refer to a hypothesis regarding the relationship between ohatsuki leaf generation and water conductance in ohatsuki shoots (Sakisaka, 1958). The information provided here will be useful for evaluating water conductance in ohatsuki shoots and leaves, which may reveal key trends in the evolution of sexual organs in terrestrial plants.

## 2 Morphology and physiology related to sexual organ development in ginkgo species

### 2.1 Ohatsuki malformation in *G. biloba*

The Mesostigmatales, chares, coleocaetales, zygnematophytes, bryophytes, protracheophytes, lycophytes, moniliphytes, and gymnophytes branches are present in this order (Wickett et al., 2014; Hasebe, 2020). In *G. biloba*, leaf development is bifurcated (Hara, 1980), and the basal body of the sperm possesses a multilayered structure (Miyamura, 2020). The former is reminiscent of the telome theory (Smith, 1955; Zimmermann, 1965; Hasebe, 2020) of protracheophytes, whereas the latter may be related to streptophyte algae (Miyamura, 2020). In the malformation of *G. biloba*, leaves bearing one or two ripe seeds on their surfaces were first found at Jyoutakuji Temple (**Supplementary Material**) in Shimoyama, Yamanashi, Japan (Shirai, 1891). As shown in **Figure 1**, these morphologies, as well as those of the leaves bearing stamens, were reported by Fujii (1896a, b, c). The words “ohatsuki icho”, which were originally expressed using Chinese characters in Japan, consists of “o” (an honorific prefix) respecting the owner of the plants, “ha” (leaf), “tsuki” (attached), and “icho” (*G. biloba*). It should be noted that Chinese characters for “icho” can mean either plants (“icho”) or seeds (“ginnan”) of *G. biloba*.

**Figure 1:**
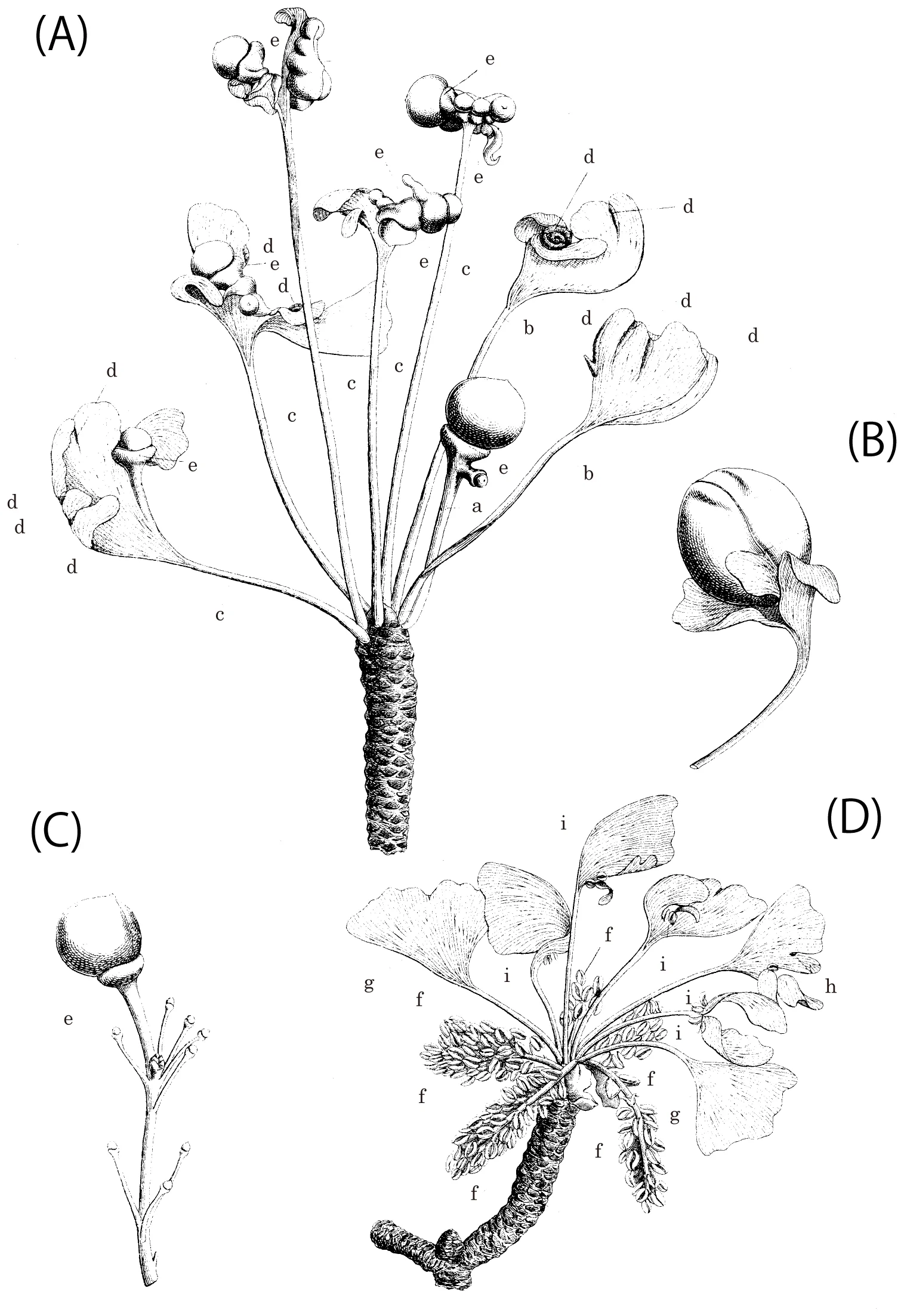
**(A)** A female short-shoot generating a normal strobilus, normal foliage, and abnormal (ohatsuki) leaves. a, normal megasporangiate strobilus; b, leaf bearing possible ovule primordia; c, leaf bearing ovules; d, possible ovule primordia; e, collar at the base of the ovule. **(B)** A foliage leaf bearing a ripe fruit. **(C)** Multi-headed strobili with a mature ovule on an apical collar and immature ovules on laterally branched stalks. **(D)** A branched short-shoot generating normal strobili, normal foliage, and abnormal (ohatsuki) leaves. f, a normal microsporangiate strobilus; g, a normal foliage leaf; h, possible stamens primordia; i, leaf bearing stamens. Adapted from Fujii (1896a, b, c), Pl. 1 in *Bot. Mag. Tokyo* 10, en7–en8, en13–en15, and en104–en110 with permission from the Botanical Society of Japan.

Female ohatsuki leaves collected at Hongokuji Temple along the Fujigawa River in Shimoyama, Yamanashi, Japan, were generated from short shoots (Fujii, 1896a, b, c). As shown in **Figures 1A** and **B**, a mature and developing seeds are attached to the surface between foliage leaf segments, whereas different developmental stages of possible epiphyllous ovule primordia are also observed in the corresponding foliage leaves (**Figure 1A**). Ohatsuki leaves that were shrunken and more segmented were generated from the same short shoot, as well as a wild-type megasporangiate strobilus. In the strobilus, one mature ovule over the collar is accompanied by a rudimentary ovule (**Figure 1A**). In contrast, multi-headed megasporangiate strobili are observed in female ohatsuki short shoots (**Figure 1C**). The morphological similarities between the ohatsuki phenotypes and ginkgo fossils can be regarded as atavism (Xing et al., 2007), although further consideration of developmental or evolutionary morphological homology is required. The number of MIKC^c^-type MADS-box genes has increased phylogenetically from Charales (Nishiyama et al., 2018) to spermatophytes (Hasebe, 2020), whereas some genes in classes A, B, D, and E are missing from the genome of *Ginkgo biloba* (Liu et al., 2021). Gene expression analyses of MADS-box genes in ohatsuki leaves are important.

At the Yamajinja Shrine in Kamiyagisawa, which is located on the opposite bank of the Fujigawa River from the female ohatsuki trees, leaves bearing stamens, normal leaves, and microsporangiate strobili were generated from a short shoot (**Figure 1D**). Some foliage leaves bore possible stamen primordia (**Figure 1D**). Male ohatsuki trees have been found at Yakuouji Temple in Ichikawamisato, Yamanashi, and in Tsukude, Shinshiro, Aichi.

Many ohatsuki trees in Japan (Ozorasha, 2009) are owned by monks and priests in their temples and shrines. Ohatsuki trees have been designated as natural heritage plants by the Japanese government (Honda, 1957) or local governments (Yamanashi Prefecture, 1931). Sampling from these plants is allowed only with the “permission for the change of the present state of natural heritages” from the government, with the agreement of the plant owners (Nagata, 2020).

### 2.2 Analysis of ohatsuki leaf generation ratio

According to Sakisaka (1958), seven long-shoots were excised from Hongokuji Temple in Shimoyama, Minobu, Yamanashi, Japan, on 20 May 1943. The majority of female ohatsuki leaves tend to be generated from short-shoots (5–7 cm long) and rarely from the shortest short-shoots (less than 1 cm long). Short-shoots on *G. biloba* street trees in Tokyo are largely less than 3 cm long. On 5 August 1943, a large branch of the Jyoutakuji Temple tree broke off because of strong winds. Of the more than 5, 000 leaves from this branch, 911 female ohatsuki leaves possessed 1–10 primordia. The number of leaves bearing one-, two-, three-, four-, five-, six-, seven-, eight-, and ten primordia were 392 (44%), 227 (25%), 132 (15%), 66 (7%), 50 (6%), 12, 6, 2, and 1, respectively. This branch bore 32 normal seeds with no leaf attachment and 23 mature ohatsuki seeds. Since 23 September 1937, 503 primordia-bearing leaves have been observed at the Hongokuji Temple. The primordia on 234 of these leaves developed into ohatsuki seeds, whereas those on the remaining 269 leaves did not.

On 27 May 1943, 14, 541 megasporangiate strobili were collected within a 1 m^2^ of the circular area under the canopy of the ohatsuki tree at Hongokuji Temple. Of these, 135 and 10, 681 were one- and two-headed (normal type) megasporangiate strobili, respectively, while 3- to 14-headed strobili were also observed. The ratio of two-headed strobili at this temple was less than 80%, whereas it was more than 90% for those at Kouenji Temple, Koishikawa Botanical Garden, and Hakusan Shrine, all of which are in Bunkyo, Tokyo. Thus, a high ratio of multi-headed, deer-antler-like megasporangiate strobili (**Figure 1C**) is characteristic of female ohatsuki trees (Sakisaka, 1958; Nagata, 2020).

### 2.3 Possible genetic background of ohatsuki shoots

Because only particular branches bear ohatsuki leaves and most of the other branches do not, Nagata (2020) presumed that certain mutation(s) had occurred in these branches to give rise to ohatsuki leaves. Referring to the legend about the origin of the ohatsuki tree at Jyoutakuji Temple, Nagata (2020) pointed out the possibility that this tree might derive from a branch from a tree in a different temple yard. A branch was excised, used as a cane by a traveling monk, and planted in a new temple. This method of propagation may have also been used in neighboring temples, resulting in several ohatsuki trees in Minobu (Sakisaka, 1958). Female *G. biloba* trees that produce larger seeds have been propagated (Crane, 2013) by grafting scions with bud sports (mutations) onto bases in different places, as has been done for other fruit cultivars in Japan (Yoshioka, 1967; Konno, 2005). Of note, one branch of this male tree was later reported to bear seeds, indicating sex conversion in one plant part (Nagata et al., 2016). Considering this sex conversion, the generation of ohatsuki phenotypes appears to be based on possible mutation(s) in sex-related genes.

In Japan, female ohatsuki icho exist in Wasada (**Supplementary Material**), Tsuruoka, Yamagata; at Inadazenbou-Sainenji Temple, in Inada, Kasama, Ibaraki; at Shirahatayama-hachimangu Shrine, in Hachiman, Mito, Ibaraki; at Fumonji Temple, in Kaminokawa, Kawachi, Tochigi; at Sugimorijinja Shrine, in Takahama, Ohi, Fukui; at Ryotokuji Temple, in Maibara, Shiga; at Reizanji Temple, in Shimotsuge, Iga, Mie; and at Kaichoji Temple in Haibara, Uda, Nara (Nagata, 2020).

In China, female ohatsuki trees have been found at Zhinudong, Daxianshan in Yiyuan (**Supplementary Material**), Shandong (Li et al., 2007); in Shaxian, Sanming, Fujian; in Jinan; in Mengyin; in Feicheng; in Xing’an, Guangxi; in Gansu; in Hunan; in Hubei and in Shaanxi (Zhou et al., 1996). A male ohatsuki tree has been reported in Doumugong, Taishan, Shandong (Xing et al., 2007).

### 2.4 Morphological comparison between ohatsuki *G. biloba* and ginkgo fossils

Three fossil species have been collected from Yima, Henan Province, China (Crane, 2013). These are *G. yimaensis* from the Jurassic formation, *Yimaia recurva* + *Baiera hallei* (two Latin names combined with “+” indicate a new single group including two original descriptions, for example, of leaves and of seeds, which were independently collected) from the Jurassic formation, and *Karkenia* sp. from the Jurassic and Cretaceous periods. In *Ginkgo yimaensis*, the seed-bearing stalks are connected to five or six seeds. These fork-shaped stalks are overlaid on two levels. The forks, known as pedicels, join another level of the peduncles. The two levels of stalks in *G. yimaensis* contrast with one level of the *G. biloba* stalk, to which one (accompanied by an abortion) or two seeds are attached. The foliage leaf of *G. yimaensis* is divided into four segments, which is reminiscent of the ohatsuki leaves of *G. biloba*, which are more segmented than the wild-type leaves (**Figures 1A, D**). In fossils of *Yimaia recurva* (Crane, 2013), a cluster of eight or nine seeds is attached to the end of the seed-bearing stalk. The foliage leaves of *Baiera hallei*, which were later regarded as belonging to the same group as the seed-bearing *Y. recurva*, are more narrowly segmented. The leaves of *K. henanensis* are apically segmented and basally joined, and the seeds of this species are clustered. Comparative morphology between the aforementioned three fossil species and extant *Ginkgo* species confirms a tendency toward chronological reduction in leaf segment numbers. The levels of seed-bearing stalks and seed numbers have been confirmed (Crane and Shi, 2020). Of note, variable levels of seed-bearing stalks in ginkgo fossils are reminiscent of the varied levels of sporangium stalk length found in telome plants (Stewart, 1983; Wellman et al., 2006; Hasebe, 2020). A fossil species of *G. apodes* has been collected (Zheng and Zhou, 2004). The morphology of this species is intermediate between *G. yimaensis* and *G. biloba*. A cluster of (up to six) ovules was suspended from one stalk level, and the leaves were flabellate and plurally dichotomous. The morphology of *G. apodes* supports the reduction hypothesis.

Leaf fossils of *Ginkgoidium nathorstii* have been collected from the Tetori Group in Japan (Yokoyama, 1890; Kimura, 1978b). Some leaves are shallowly divided into two segments, and the lower parts are ovoidal, similar to the extant species *G. biloba*, whereas some *G. nathorstii* leaves are not segmented and resemble arrows. *G. yimaensis* and *G. apodes* typically have deep leaf segmentation at the base, distinguishing four or five segments, more than the number of segments (1 or 2) found in *Ginkgoidium nathorstii*. Leaf fossils of this species found alongside the seeds have recently been collected by the Tetori Group (Matsuoka, 2000; Yasuno, 2007). One specimen was found at Kuwajima (**Supplementary Material**), Hakusan (SBEI P-3863, Shiramine Institute of Paleontology, original spelling, *Ginkgoidium Nathorsti*), and another at Miyama, Fukui (FCMNH-GF6269, Fukui City Museum of Natural History). The former contains four non-segmented leaves and one two-segmented leaf, along with eight mature seeds suspended by a long pedicel generated from a short shoot (Matsuoka, 2000; Sekido, 2002). The latter includes seven or eight leaves, two of which are two-lobed. The others are not segmented and have four or five cavities, presumably depressed by seeds (Yasuno, 2007). A leaf fossil of a related species, *Ginkgoites digitata* (GT090, Komatsu City Museum), has also been collected by the Tetori Group. The genus *Ginkgoites* has been defined in comparison with *Ginkgo* (Matsuo, 1962).

In addition to macromorphology, the stomatal micromorphological features of each ginkgo fossil may be characteristic. The cuticle layer surrounding the plant prevents water transpiration from the inside (Terashima, 2013). This part of the plant body is often stably retained in fossils that clung to coral material. This layer can be dissolved using an acid solution and then neutralized using an alkaline solution to generate cuticle layer fossil specimens, which can be observed under light and electron microscopy. The stomata of *G. yimaensis* are larger (150–175 × 100–125 µm) and are accompanied by six to eight subsidiary cells, while those of *G. biloba* are monocyclic and much smaller (71–124 µm) (Zhou et al., 2012). The larger diameter of the *G. yimaensis* fossil species than that of *G. biloba* (Rudall et al., 2012) may reflect different rates of water transpiration from leaves as well as different levels of photosynthesis, although the climate (Kimura, 1978a) and ambient air composition (Terashima, 2013) during the Jurassic period were different from current conditions.

### 2.5 Evaluation of the influence of water conductance in ohatsuki shoots and leaves

In 1896, Fujii grafted shoot scions bearing ohatsuki leaves, which were obtained from Hongokuji Temple in Shimoyama, Yamanashi, onto a stock in Koishikawa Botanical Garden, Tokyo. More than 60 years later, their offspring had grown into independent trees that had never borne ohatsuki leaves (Sakisaka, 1958). Over the following 60 years, no ohatsuki leaves were observed on these trees. These findings suggest that the expression of genetic factors in ohatsuki shoots is only possible under specific environmental water conditions. Sakisaka (1958) hypothesized that the generation of ohatsuki leaves was due to the trees receiving a greater water supply than they transpired through their leaves in Shimoyama, whereas this environment was not replicated in Koishikawa. This hypothesis was partly based on the observations of two-segmented leaves in shaded areas and non-segmented leaves in sunny areas of a botanical garden in Melbourne. More water evaporates from leaves in sunny areas than from those in shaded areas. Sakisaka (1958) also noted that some trees growing in windy areas bore small leaves. However, neither water transpiration from ohatsuki leaves nor water uptake from ohatsuki tree roots has been demonstrated. Strong seasonal southern winds (Yamanashi Prefecture, 1931; Mochizuki, 2015) shake the branches of ohatsuki trees vigorously, increasing the evaporation from their leaves.

The amount of water transported by an individual plant is defined as the value obtained by multiplying the leaf transpiration conductance by the difference in water potential between the soil and the leaf (Taneda et al., 2016). The thickness of the leaf boundary layer is determined by the square root of the value obtained by dividing the leaf width by the wind speed (Terashima, 2013; Jones, 2014). By measuring stomatal conductance, the water vapor concentration inside the leaves, the water vapor concentration on the leaf surface outside the stomata, and water transpiration from ohatsuki leaves (**Figures 1A, D**), the transpiration rate of ohatsuki leaves may be directly evaluated in the future. According to a recent report, salt stress in *G. biloba* trees results in lower stomatal density and a smaller proportion of xylem conduction (Matsuura et al., 2026). When the night/day temperature regime of 20/25°C shifts to 30/35°C, stomatal density increases; however, stomatal size, stomatal pore length, and the stomatal index value (stomata counts/total epidermal cells) decrease (Carli et al., 2025). These results indicate that stomatal number, density, and stomatal conductance are influenced by the environment. It is important to integrate these modern studies into future physiological experimental designs.

## 3 Conclusion

A comparison of the morphologies of ginkgo fossils and extant *Ginkgo* species suggests three evolutionary trends. There have been reductions in the number of leaf segments, the number of seeds, and the levels of seed-bearing stalk forks. The malformed morphologies of the male and female ohatsuki leaves in *G. biloba*, its more segmented leaves, and its multi-headed, deer-antler-like megasporangiate strobili are similar to those of some ginkgo fossils. These morphological similarities can be regarded as atavisms in ohatsuki *G. biloba*. MADS-box genes are important for evaluating these morphological similarities. Shoots with bud sports that generate ohatsuki leaves in certain temples or shrines may have been derived from scions that were later grafted onto stocks in other religious sites to generate new ohatsuki trees. Future physiological experiments using recent technologies with portable instruments to measure stomatal conductance will be required to evaluate the hypothesis that water conductance through ohatsuki shoots and leaves, which may be affected by seasonal winds, is due to the generation of ohatsuki leaves. Molecular biological analyses are promising for these experiments.
