# Supplementary material for: A review of the morphology of ohatsuki (stamen- or ovule-bearing) leaves, with relevance to physiological analyses of Ginkgo biloba
Source: Front Plant Sci. 2026 Oct 29;17:1983013. doi: 10.3389/fpls.2026.1983013 (PMC13760444; doi:10.3389/fpls.2026.1983013)
Supplement: Supplementary file 1 [file Table1.pdf]

Supplementary Material

Places expressed in English, Chinese, and Japanese

Doumugong, Taishan, Taian, Shandong	山东省泰安市泰山斗母宫	山東省泰安市泰山斗母宮
Feicheng	肥城	肥城
Fumonji Temple	普门寺	普門寺
Gansu	甘肃省	甘粛省
Hunan	湖南省	湖南省
Hubei	湖北省	湖北省
Hongokuji Temple	本国寺	本国寺
Inadazenbou-Sainenji Temple	稻田禅坊西念寺	稲田禅坊西念寺
Jinan	济南	済南
Jyoutakuji Temple	上泽寺	上沢寺
Kaichoji Temple	戒长寺	戒長寺
Kouenji Temple	光圆寺	光円寺
Kuwajima, Hakusan, Ishikawa	石川县白山市桑岛	石川県白山市桑島
Mengyin	蒙阴	蒙陰
Miyama, Fukui, Fukui	福井县福井市美山町	福井県福井市美山町
Ryotokuji Temple	了德寺	了徳寺
Reizanji Temple	零山寺	零山寺
Shaxian, Sanming, Fujian	福建省三明沙县	福建省三明沙県
Shirahatayama-hachimangu Shrine	白幡山八幡宫	白幡山八幡宮
Sugimorijinja Shrine	杉森神社	杉森神社
Shaanxi	陕西省	陝西省
Tetori Group	手取层群	手取層群
Wasada, Tsuruoka, Yamagata	山形县鹤冈市早田	山形県鶴岡市早田
Xing'an, Guangxi	广西省兴安县	広西省興案県
Yima, Henan	河南省义马	河南省義馬
Zhinudong, Daxianshan, Yiyuan, Shandong	山东省沂源县大贤山织女洞	山東省沂源県大賢山織女洞
Zhuanchengzi	砖城子	磚城子
